# Lunches in UK early years education and care settings: cross-sectional analysis of food processing, provision and intake

**DOI:** 10.21203/rs.3.rs-9452819/v1

**Published:** 2026-04-23

**Authors:** Rachel Maishman, Zoi Toumpakari, Amy Turner, Marie Murphy, Corby K. Martin, Miranda Pallan, Kim Hannam, Peter S Blair, Sharon A Simpson, Laura Johnson, Ruth Kipping

**Affiliations:** Bristol Medical School, University of Bristol, Bristol, UK; School for Policy Studies, University of Bristol, Bristol, UK; Bristol Medical School, University of Bristol, Bristol, UK; Department of Applied Health Sciences, College of Medicine and Health, University of Birmingham, Birmingham, UK; Pennington Biomedical Research Centre, Baton Rouge, USA; Department of Applied Health Sciences, College of Medicine and Health, University of Birmingham, Birmingham, UK; Bristol Medical School, University of Bristol, Bristol, UK; Bristol Medical School, University of Bristol, Bristol, UK; MRC/CSO Social & Public Health Sciences Unit, University of Glasgow, Glasgow, UK; Bristol Medical School, University of Bristol, Bristol, UK; Bristol Medical School, University of Bristol, Bristol, UK

**Keywords:** Early Childhood Education and Care, lunch, serving size, ultra-processed foods, UPF, foods high in salt fat and sugar, HFSS, non-core foods

## Abstract

**Objective:**

Most young children attend early childhood education and care (ECEC), but little is known about lunch provision, intake and processing. We investigated lunch quality, serving size and consumption within ECECs by provision (packed vs. ECEC provided) using the UK nutrient profile model (high fat, salt or sugar, HFSS), ultraprocessing (UPF) and non-core as food quality measures.

**Design:**

Lunches of children attending ECEC were analysed using food photography. Total kcal served/consumed and % total kcal from foods classified as HFSS, UPF or non-core were calculated. Multivariable mixed effects linear regression modelled associations of serving size with provision or quality adjusting for age, zBMI and deprivation.

**Setting:**

ECEC providers in England and Scotland.

**Participants:**

532 children aged 2–4 from 52 ECECs.

**Results:**

Packed lunches were more prevalent in England (48%) than Scotland (3%). Packed lunch servings were larger (adjusted mean difference (aMD) 120.0kcal (95%CI 76.7 to 163.3), p<0.001) but consumed kcal were not (aMD 16.0 (−28.0, 60.0), p=0.476). HFSS, UPFs and non-core food servings were 15–24% higher in packed compared with ECEC provided lunches. Overall, serving size was associated with kcal consumed: for every 100kcal served 60kcal (56, 69) were consumed. Higher HFSS and non-core food provision resulted in increased consumption.

**Conclusions:**

Servings in packed lunches were larger and poorer quality, with both features associated with consuming more. We reinforce guidance to avoid serving HFSS food and provide small servings initially, to allow children to eat optimal energy intakes. Universal free lunches in ECECs could improve lunch quality and serving sizes.

## Introduction

Childhood obesity is a global public health challenge, with over 5% of children under five years living with obesity^(1)^ in 2022. In England and Scotland, the proportion of children aged 2–6 living with obesity in 2023/2024 was 22.1%^(2)^ and 17%^(3)^, respectively. On average, 76% of three-year-old children^(4)^ attend early childhood education and care (ECEC) in Organisation for Economic Co-operation and Development (OECD) countries. Demand for ECEC services, such as nurseries or preschools, is high in England and Scotland ^(5),(6)^. The recommended daily kcal consumption for children aged 1–4 is 1108 kcal, with the majority (369) consumed at lunch time^(7)^. Eating occasions in childcare settings are associated with 15% larger portion sizes, thus ECECs represent a high-risk environment for passive over-consumption^(8)^. Over-consumption can derive from large portion sizes and high energy density meals served in ECEC settings^(9, 10)^. Previous studies established that portions sizes consumed in ECEC settings exceeded recommendations^(10, 11)^, however they did not measure both served and consumed portion sizes leaving it unclear if servings translate into excessive consumption or food waste.

Whilst there is food provision guidance for ECECs in England^(7, 12)^, there are no government mandated nutritional standards^(13)^, and many children have ‘packed lunches’ provided from home. Scotland, however, has national food standards that all ECEC must adhere to and provision of universal free meals to children attending ECEC^(14)^. In schools, lower energy intake was reported from school-sourced food (“school meals”) compared to home-sourced food (“packed lunches”) in UK children aged 4–10 years^(15)^, however comparable intake in ECECs has not been studied.

Food quality has been measured using various models, including the UK nutrient profile model^(16)^ that identifies foods that are high in fat, salt or sugar (HFSS) and the Australian dietary guidelines which define foods as core (provides some essential nutrients) or non-core (surplus to nutritional requirements)^(17)^. More recently, quantifying the consumption of ultra-processed foods (UPFs), which have undergone industrial processing and contain numerous preservatives and other additives, has been explored. UPFs are hyper-palatable, chemically modified, convenience foods including snacks, drinks, ready-to-eat meals and other food products^(18)^. Consumption of UPFs, HFSS and non-core foods are associated with adverse health outcomes such as cardiovascular disease, greater mortality risk, obesity, and other non-communicable diseases (NCDs)^(19)^.

Whilst UPFs are often high in fat, sugar, and salt, not all UPF foods are classified as HFSS^(20)^. Studies have found UPFs contribute between 20–47% of total daily energy in children under two years^(21)^, but the most common UPFs were yoghurts, high-fibre breakfast cereals and wholegrain breads^(21)^, suggesting that UPFs in this age group are not necessarily HFSS foods. It remains unclear how much UPF overlaps with food quality measures based on existing dietary guidelines, or whether UPF are problematic over and above their HFSS content.

UPF is commonly provided in UK primary and secondary schools^(22)^, and non-core foods have been shown to make up 44.3% of the mean energy intake in ECECs for children aged 1.5–3 years^(15)^. Despite this, research has shown that UPF and non-core food consumption is lower in the school environment compared to at home and eating with family^(23)^. ECEC studies in England have found that food provided contained higher than recommended energy, fat, sugars, and salt for early years children, and was deficient in iron and zinc micronutrients^(24)^. Evidence has shown packed lunches for children generally have poor nutritional quality; one study conducted in Australian ECEC found that the average packed lunch contained over three times the Australian Dietary Guideline’s daily recommendation for non-core foods for early years^(25)^. This finding is consistent with findings from UK primary and secondary schools that found packed lunches contained more UPFs and less vegetables than meals provided by schools^(22, 26)^, however differences in quality have been shown to diminish at the secondary school level where poor lunch quality was demonstrated irrespective of provision type^(15)^. There is a gap in understanding the impact of UPF, HFSS, and non-core food content in packed lunches and related consumption in early years children.

Excessive serving sizes also lead to passive overconsumption in part because of the high energy density of HFSS foods, UPF and non-core foods^(9)^. However, larger portion sizes are also linked to greater energy intake independent of food quality in young children^(9)^. We hypothesised that packed lunches contain a higher % of kcal served from HFSS/UPF/non-core foods that leads to a larger total lunch serving and increased kcal consumption in children. We aimed to investigate whether packed lunches contained larger servings of poorer quality food (assessed by % of total intake from foods HFSS/UPF/non-core foods) and if this was associated with greater energy intake in ECEC settings, as well as whether food quality mediated the relationship between food provision and lunch consumption. A secondary aim of this study was to explore how the three food classification methods (UPF, HFSS, and non-core) overlap for foods consumed in ECEC.

## Methods

### Study design

This study used baseline measures collected between June 2022 and February 2023 for the NAPSACC UK multicentre cluster randomised controlled trial (2022–2024), which aimed to reduce energy consumption and increase physical activity levels in ECEC settings for 2–5 year olds^(27)^. Follow-up trial data were not included because of the exposure of half the ECEC settings to an intervention designed to improve nutrition.

#### Recruitment

ECEC providers (private or local government nursery schools, nursery classes or pre-schools) from four local government areas (LGAs) across the UK (Somerset, Swindon, and Sandwell in England, Arran and Ayrshire in Scotland) were invited to participate in the study. Children were recruited from consented ECEC settings through opt-in consent obtained from parents/carers. Children were eligible if they were: at least 2 years old at the time of data collection; not attending primary school; attending the ECEC setting at least 12 (full year attendance) or 15 (term-time only attendance) hours/week; and consumed lunch at the ECEC setting at least once a week (ECEC or parent provided which we term ‘packed’ lunch). Consented children who contributed ECEC lunch data at baseline were eligible for inclusion.

#### Diet assessment and processing

The Remote Food Photography Method (RFPM) was used to measure food served and consumed in lunches within the ECEC setting (further details in Appendix page 1)^(28)^. Food composition data from the latest UK National Diet and Nutrition Survey (NDNS) were used to compute energy (kcal) served and consumed for each food/drink item. Each item was classified as core/non-core^(29)^ (used in the main trial protocol), assigned a NOVA classification building on previous work in the UK NDNS^(22)^ (NOVA 1 to 4, classified as UPF [NOVA 4] or non-UPF [NOVA 1–3]), and scored according to the UK Nutrient Profile Model^(16)^ (classified as HFSS [score ≥ 4 for food or ≥ 1 for drinks] or non-HFSS). Classifications according to HFSS, UPF and non-core are given in Appendix Table 6.

For each child, kcal served and consumed across all food/drink items were summed to derive the total kcal served and consumed at lunch. Total kcal served and consumed from HFSS, UPF and non-core foods were summed and expressed as the proportion of the total kcal served or consumed, respectively.

#### Potential confounders

Body Mass Index (BMI) (kg/m^2^), converted to centiles (z-BMI) using the UK 1990 growth reference chart^(30)^, was classified as healthy weight (zBMI < 1.04; 1.04 = 85th centile) or unhealthy weight (zBMI ≥ 1.04). Parents/carers reported demographic information (child’s date of birth, sex and home postcode) at recruitment. Child deprivation level was based on the child’s postcode using the Index of Multiple Deprivation (IMD)^(31)^ or Scottish IMD (SIMD)^(32)^ for children attending ECEC providers in England and Scotland, respectively. Deprivation was categorised as high (deciles 1–3 of IMD/SIMD), moderate (deciles 4–7) and low (deciles 8–10). Season was categorised as Winter/Spring (December-May), Summer (June-August) and Autumn (September-November).

### Statistical analysis

Analyses were carried out across eight outcome measures: total kcal served, total kcal consumed, proportion of total kcal i) served and ii) consumed from: HFSS foods, UPFs and non-core foods. Outcome measures were summarised across child and ECEC provider characteristics using the median and interquartile range (IQR) due to skewed distributions. In all association models, ECEC provider was fitted as a random effect to account for clustering. Unadjusted associations of lunch provision (packed vs. ECEC-provided; key exposure) and potential confounder variables (age, gender, zBMI [healthy/unhealthy weight], child deprivation (high/moderate/low), provider type (attached/not attached to school), LGA, country, season of data collection) with each outcome measure were modelled using univariable mixed-effects linear regressions. Multivariable mixed-effects linear regression models were fitted for each of the eight outcome measures with lunch provision (main exposure) and adjustment for all potential confounder variables; LGA was also fitted as a random effect due to correlation with lunch provision, age, child deprivation, season of data collection, and provider type.

### Mediation analyses

**Figure 1:**
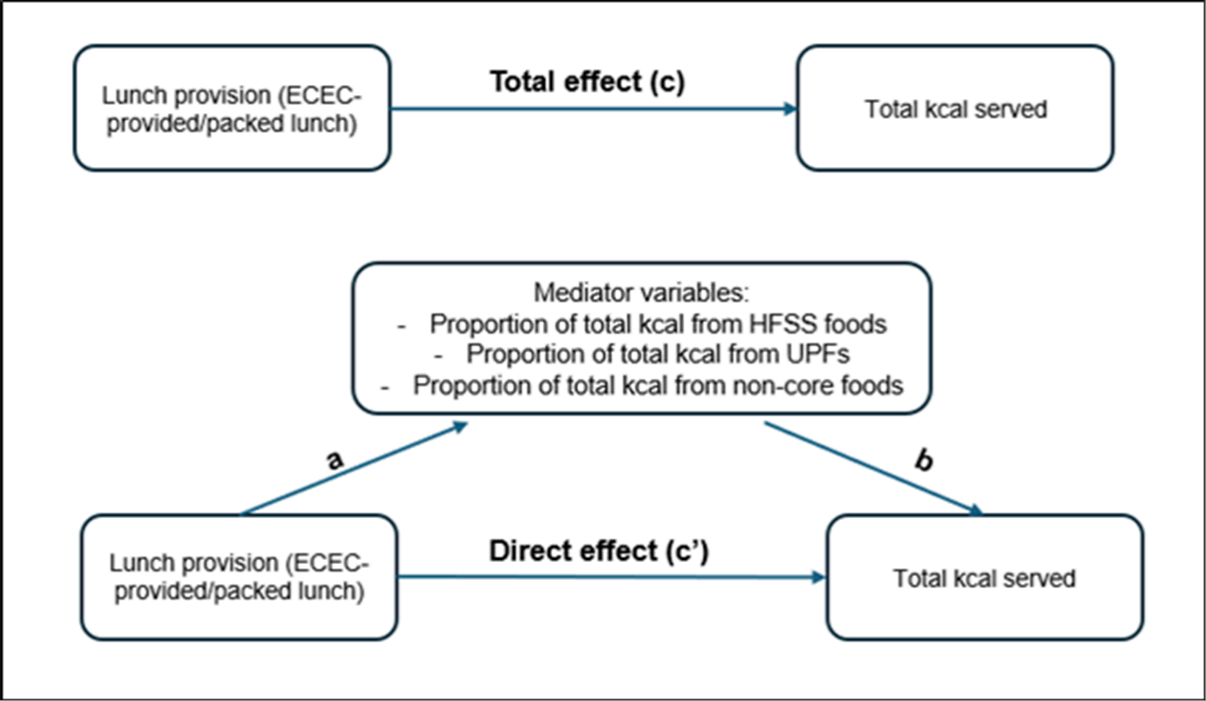
Mediation analyses to explore the extent to which food quality mediates the relationship between lunch provision and total kcal served. Abbreviations: ECEC - early childhood education and care; HFSS - high fat, salt or sugar; UPF – ultra-processed food.

Mediation analyses were performed to assess the extent to which the effect of lunch provision on serving size is mediated by food quality in ECEC-provided/packed lunches (Fig. 1). Details on mediation analyses are given in Appendix page 1.

#### Sensitivity analyses

Analyses were repeated on the subset of children attending ECEC providers in England due to the aforementioned differences in food provision between England and Scotland. Further sensitivity analyses were carried out on the subset of children consuming ECEC provided meals to compare the size and quality of ECEC provided meals between England and Scotland; in these analyses, country was fitted as the exposure variable in place of lunch provision.

#### Comparison of different food classification methods

The relationships between different classification methods were explored using food-level analyses. The proportion of foods classified as HFSS, UPF and non-core out of all unique food items, as well as cross-tabulations of the three, were described; each food item was included once irrespective of the number of times the food was recorded in the dataset. Main food groups were summarised for food items within different combinations of the classification methods.

Analyses were performed using Stata v18.5.

## Results

### Participant characteristics

A total of 532 children aged 2–5 were included; over 80% were three or over and equal numbers were male and female. A quarter of children were living with overweight or obesity and 80% came from areas of high or moderate deprivation. Two thirds attended ECEC providers in England, and 60% of these (217/359) attended ECEC providers in Somerset. Greater deprivation was observed in Sandwell (Sandwell 75% high deprivation; Ayrshire and Arran 44%; Swindon 29%; Somerset 17%) (Table 1). Data collection in Ayrshire and Arran was primarily carried out in Autumn; in other LGAs it was spread across seasons.

### ECEC provided lunches vs. packed lunches

Two thirds of children had ECEC provided meals, but a greater proportion was observed in Scotland (97%) than England (52%). The proportion of children with packed lunches (n = 177/532) varied across LGA; 63% of children in Swindon, compared to 49%, 35% and 3% in Somerset, Sandwell and Ayrshire and Arran, respectively (data not shown). Fewer packed lunches were observed in Autumn (24% compared to 41% in Winter/Spring and 36% in Summer), which corresponds to data in Ayrshire and Arran being predominantly in Autumn and the low frequency of packed lunches in this area.

### Packed and ECEC provided lunches settings in England and Scotland combined

Children were served a median 433.9 kcal (IQR 339.9 to 564.1) at lunch in this study. Packed lunches served more kcal than ECEC provided meals (median 516.2 kcal served compared to 403.3 kcal, p < 0.001). More kcal were served in England than Scotland (median 466.1 kcal compared to 382.9 kcal, p < 0.001); kcal served also differed across LGA within England, with fewer kcal served in Sandwell, the area with the lowest proportion of packed lunches, compared to Somerset and Swindon. (Table 1).

Children consumed a median 319.5 kcal (IQR 214.4 to 439.4) at lunch. Despite the larger serving sizes of packed lunches, there was no evidence that kcal consumed differed by food provision (median 306.1 kcal ECEC provided; 359.6 kcal packed lunches, p = 0.110) (Table 1). Consumed lunches were smaller in Scotland (median 287.7 kcal in Scotland; 336.6 kcal in England, p = 0.033), with further differences seen between LGA in England (median 231.1 kcal consumed in Sandwell compared to 359.6 kcal and 385.9 kcal consumed in Somerset and Swindon, respectively), mirroring trends seen in kcal served. Fewer kcal were consumed in Autumn (median 301.8 kcal) compared to Summer (319.0 kcal) and Winter/Spring (342.2 kcal), p = 0.017.

The median proportion of total kcal served in lunches from HFSS, UPF or non-core foods were 48.3%, 57.9% and 37.9%, respectively (Table 1). Compared with ECEC provided lunches, packed lunches had a higher proportion of kcal served and consumed from HFSS foods, UPFs and non-core foods (Table 1 and **Appendix** Table 1). A lower proportion of kcal were served from HFSS foods in children under three (median 40.4% compared to 50.2% and 51.6% in children aged 3–4 and > 4, respectively, p = 0.044), however more children under 3 had ECEC provided lunches (76% of children < 3 years compared to 67% of children aged 3–4 and 60% of children aged > 4). Children from low deprivation areas had a smaller proportion of the kcal served and consumed from HFSS foods (low deprivation: median 42.1% served and 42.9% consumed; moderate deprivation: median 50.1% served and 48.8% consumed; high deprivation: median 48.9% served and 44.8% consumed; p = 0.019 for served, p = 0.033 for consumed).

### Multivariable associations

In adjusted analyses, packed lunches were associated with higher kcal served (adjusted mean difference (aMD) 120.0 (76.6, 163.3), p < 0.001) but not kcal consumed (aMD 16.0 (−28.0, 60.0), p = 0.476). Food quality was worse with packed lunches; UPFs, HFSS and noncore foods contributed between 15% and 24% higher kcal served, and between 11% and 21% higher kcal consumed (Table 2).

Regardless of lunch provision, total energy consumed was associated with: kcal served (0.6 (0.56, 0.69) kcal consumed for each extra 1 kcal served); the proportion of total kcal served from HFSS foods (1.5 (0.9, 2.0) kcal consumed for each 1% extra kcal served from HFSS foods equivalent to a 15 (9, 20) kcal higher intake for a 10% increase in HFSS food); and the proportion of kcal served from noncore foods (0.8 (0.3, 1.4) kcal consumed for every 1% extra kcal served from non-core foods). There was no evidence of an association between kcal consumed and kcal served from UPFs (Table 2).

The association of packed lunches with kcal served was partially mediated by the increased kcal from HFSS served in packed lunches (direct effect of packed lunches aMD 67.5 kcal (21.5, 113.6), p = 0.004 after controlling for the proportion of kcal served from HFSS foods; indirect effect representing a 44% mediation of the total effect). There was no evidence of mediation for the proportion of the total kcal served from UPFs or non-core foods on the association between packed lunches and larger lunch servings (Table 3).

### Packed and ECEC provided lunches settings in England

Similar to analyses in the whole cohort, when restricting the sample to England, packed lunches contained more energy and poorer quality food than ECEC provided lunches (**Appendix Tables 2 and 3**). In contrast to the whole cohort, lunches in English ECECs attached to schools had a higher proportion of the total kcal served and consumed from HFSS foods (**Appendix Tables 2 and 3**). Multivariable results stratified by country were consistent with those including ECEC providers in both England and Scotland (Table 2).

### ECEC provided lunches in England and Scotland

ECEC provided lunch servings were 403.3 kcal (304.7, 523.9), while consumed kcal were 306.1 kcal (199.4, 413.7) (**Appendix Tables 4 and 5**). Neither kcal served or consumed in ECEC provided lunches differed between England and Scotland before or after adjustment (Table 2, and **Appendix Tables 4 and 5**). Food quality did not differ by country or any other child/ECEC provider characteristics in univariable analyses (**Appendix Tables 4 and 5**). Consistent with other analyses presented, kcal served and the proportion of kcal served from HFSS and non-core foods were associated with kcal consumed in ECEC provided lunches (Table 2); a larger increase in kcal consumed was seen for each kcal served in analyses restricted to ECEC provided lunches (0.8 (IQR 0.7, 0.9) kcal consumed for each kcal served) compared to analyses including packed lunches, suggesting lower waste from ECEC provided meals.

### Comparison of food classification systems

**Figure 2:**
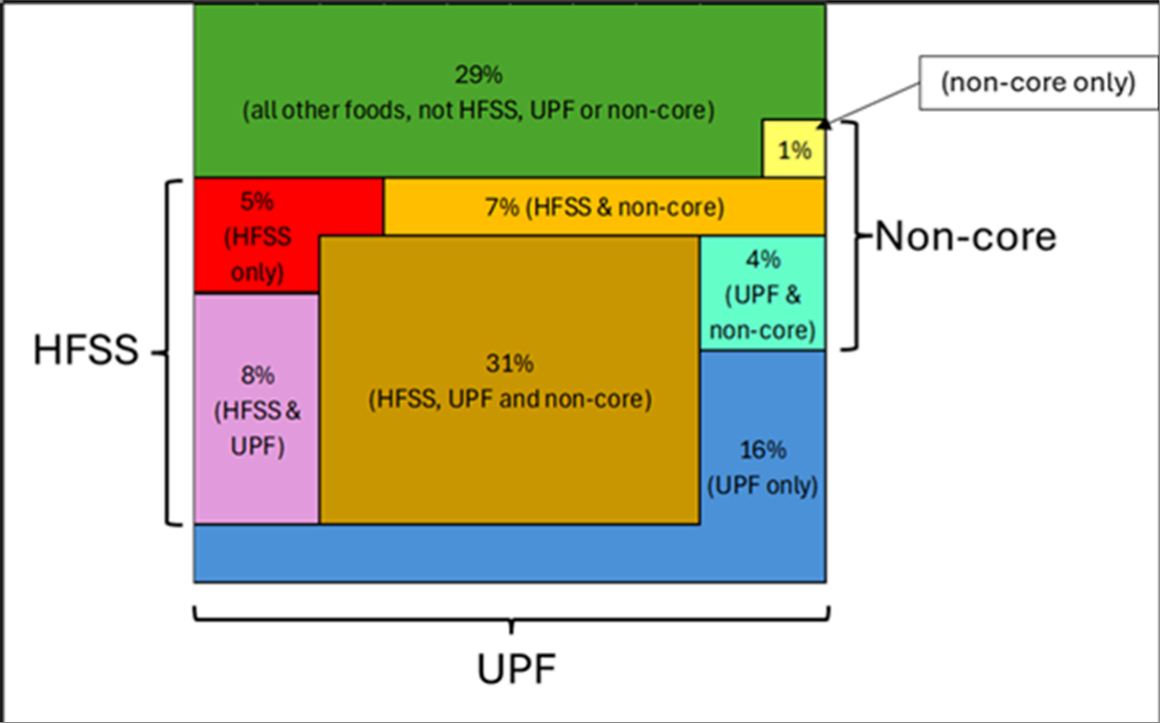
Proportion of food items classified according to each of the three classification methods (high fat, salt or sugar [HFSS], ultra-processed foods [UPF] and non-core) within the NAPSACC UK dataset

Across the 532 children included in this study, 551 unique food/drink items were recorded; 50% were HFSS, 59% were UPF and 42% were non-core (Fig. 2). Thirty-one percent of food/drinks were classified as HFSS, UPF and non-core. Most non-core foods were also identified as HFSS, UPF or both (228/231, 99%). In contrast, 27% of foods were UPF but not identified as HFSS or non-core, and 9% of foods were identified as HFSS but not UPF or non-core. Just under one third of foods were neither HFSS, UPF or non-core.

Main food groups were summarised for each food within each combination of classification methods (Table 4). The majority of foods classified as HFSS, UPF and non-core were biscuits (24%), buns/cakes/pastries/fruit pies (21%), or crisps and savoury snacks (14%). In contrast, foods classified as neither HFSS, UPF nor non-core were mainly fruit or vegetables (50%). Foods classified as UPF but not HFSS or non-core included yogurt-based desserts (15%), cooked vegetables (11%) and white bread (10%). Core food items that met HFSS and UPF criteria were most frequently cheese (20%), coated chicken (11%) and pasta/rice/other cereals (11%). Almost half the foods classified as HFSS and non-core but not UPF were buns/cakes/pastries /fruit pies, representing sweet desserts that were homemade within the ECEC providers.

## Discussion

Our study is the first to describe how packed vs. ECEC provided lunches vary in quality and portion size (served and consumed) by children in ECEC in England and Scotland. Regardless of lunch provision, larger servings and more HFSS food served at lunch were associated with consuming larger lunches, reinforcing government advice to avoid serving HFSS food and to start meals with small servings, allowing children to ask for more if they are still hungry to ensure optimal energy intakes in ECECs^(33)^. Using three classifications of food quality, we confirmed our hypotheses that packed lunches were poorer quality and contained more HFSS, UPF and non-core foods than ECEC provided lunches. Defining lunch quality by HFSS, and to a lesser extent non-core foods, poorer quality lunches were associated with the larger total serving size of packed vs. provided lunches. While packed lunches contained as much as 74% total energy from UPF, defining quality this way was not associated with larger total servings or consumption, leaving the importance of processing beyond the nutrient content of food unclear in terms of preventing passive overconsumption at young ages.

In our study, the average kcal consumed at lunch (median 320 kcal) was similar to that reported in a representative sample of UK children aged 1.5–5 years old (median ~ 286 kcal)^(8)^. Although our study assessed only one meal, it is in accordance with previous research showing that contribution of UPFs to daily energy intake was 61% among UK children aged 2–5 years old^(34)^. In contrast, evidence from a British twin cohort, showed that at 21 months (n = 2,591) contribution of UPF to daily energy intake was lower to our study (46.9%) but increased to 59.4% at 7 years^(21)^. Similar to our findings, yoghurts have also been reported as the most common UPF at 21 months^(21)^.

We demonstrated higher kcal served in packed lunches compared to ECEC provided lunches, but no difference in kcal consumption suggesting greater food waste from packed lunches. Larger packed lunch portion sizes may be because parents are unaware of the level of food waste if disposed of by ECECs, concerns over children not eating enough or a desire to give children choice. In contrast, less food waste in ECEC provided meals may be attributed to ECEC staff having a greater awareness of portion size guidelines or self-serving of food by children.

We found that overall packed lunches contained more HFSS, UPFs, and non-core foods than ECEC provided lunches. This is consistent with a study in older children that found that confectionery and savoury snack foods, high in fat, sugar, or salt, were consumed more frequently from packed lunches than meals provided in UK schools^(26)^. One US study in ECECs found a large proportion (25%) of packed lunches contained fruit drinks high in sugar and this contributed to a poorer Healthy Eating Index-2020 (HEI) score^(35)^. Interventions designed to improve nutritional quality of packed lunches in ECECs have had varied success^(36)^. The ‘Lunch is in the Bag’^(37)^ intervention based in US ECECs was effective at improving vegetable and wholegrain content in packed lunches, however parents continued to provide high proportions of HFSS and non-core foods in packed lunches. Evidence suggests that the quality of packed lunches in ECECs cannot easily be improved through intervention^(36)^, and consequently lunches provided by ECECs are more likely to ensure healthy food provision. ECEC provided lunches did however still contain large quantities of UPFs, HFSS and non-core foods, and further guidance/mandated standards to reduce these in ECEC provided lunches is needed.

Our analysis of ECEC provided meals found that food quality did not differ by deprivation, suggesting that ECEC provided lunches may help reduce health inequalities. This is consistent with the evaluation of the introduction of universal infant free school meals for children aged 4–7 in primary schools in England and Scotland in 2015, which found children ate less UPFs with the greatest effects for children from low-income families^(38)^. Further, this evaluation found a 33% increase in uptake^(39)^, again with the greatest uptake being in low-income families demonstrating the potential to reduce health inequalities.

We found that larger proportions of kcal from HFSS and non-core foods were associated with higher kcal consumption, consistent with a previous study that found larger portions of foods such as pizza and sugar-sweetened beverages that are classified as HFSS and non-core foods, were associated with higher energy intake in children^(40)^. The association that we observed between HFSS food servings and larger lunch servings is a possible explanation for the higher consumption at lunch, in line with experimental evidence that serving larger portions drives higher intakes^(9)^. The higher energy density of HFSS foods may be another independent explanation for association between HFSS servings with increased lunch consumption^(10)^. Furthermore, studies have found that the hyper-palatability of HFSS, UPFs, and non-core foods drive increased energy intake and portion size by eliciting a brain reward response which can become hyper-sensitive^(41)^. This may have greater implications for children in areas of higher deprivation as it has been generally established that greater deprivation is associated with poorer diet quality in children^(42)^.

The proportion of kcals from UPFs was not associated with kcal served or consumed in our study, in contrast to the evidence suggesting that it promotes overconsumption^(41, 43)^; there may be distinct differences in the types of UPF commonly consumed by this age group (both in packed lunches and ECEC provided lunches). While most HFSS foods and non-core foods were also classified as poor quality according to at least one other classification system, almost 30% of UPFs were neither HFSS or non-core; compared to a sample of UK adults and children^(20)^, our findings showed a higher percentage of foods being UPF only (27% vs. 16%). This was mainly due to the high frequency of foods such as yogurt/fromage frais/dairy desserts, bread and cooked vegetables (including baked beans) in this age cohort. Yoghurts/fromage frais have been previously reported among the 10 highest food contributors to UPFs in the UK^(20)^. They are an important source of calcium and are recommended for children aged 1–5 years, as long as they are unsweetened^(44)^.

In our findings, white bread was also among the high contributors to UPF only foods; although evidence in the association between white bread and disease risk is mixed^(45)^, studies have shown that white bread may be more detrimental to health, such as obesity and cardiovascular disease compared to wholegrain bread^(46)^. Healthier swaps, such as unsweetened yoghurts and wholemeal bread, are encouraged^(44)^, however these are also UPFs. ECEC settings, as well as families, face substantial time and resource constraints, hence using only unprocessed or minimally processed foods may present a challenge to financial and time budgets. Future research should explore differences in specific food items provided in packed lunches and ECEC provided meals.

A strength of the study is the inclusion of a novel diet data collection methodology that enabled direct estimations of, rather than proxy-reported, foods served and consumed within the free-living environment of ECECs. Whilst the methodology is high-burden for fieldworkers to ensure all food is captured and coded accurately and has the potential for behaviour modification, it removes the risk of recall bias. The study included ECEC providers and children from a broad range of deprivation and geographical areas. Including ECEC settings from England and Scotland enabled a comparison of food quantity and quality between two countries with different lunch provider policies (Scottish government funded lunches) adding strength to the understanding of different policies.

A limitation is the analysis of lunch data only; snacks provided within the ECEC setting were not included due to the heterogeneity in provision, with considerable differences in timings and the way snacks were served. Snack data were included in the analyses for the main trial^(47)^ and the findings demonstrated that snacks were predominantly healthy and in-line with guidelines limiting the potential for further insights into variation in poor quality or larger servings of ECEC food. It is possible that some of the excess kcal served in packed lunches may have included additional snacks intended for consumption later in the day. As all food served in a lunch box was considered by fieldworkers as intended for consumption at lunch, then any food intended for consumption later in the day may have been misclassified as lunch and incorrectly interpreted as food waste in our findings. Other meals consumed within the ECEC settings (breakfast and tea/dinner) were not collected; in the NAPSACC UK pilot study, 94% of children were present for lunch, while 26% had breakfast in the ECEC setting and 23% had dinner. Furthermore, food intake outside of ECECs, that accounts for up to 80% of eating occasions in this age group, has not been collected therefore we can’t estimate compensatory eating behaviours. The NDNS, which has 4 days of intake across all eating locations has previously highlighted that food intake at ECECs may be larger^(8)^ and poorer quality than food eaten at home^(15)^. Our data combining data on both servings and consumption, objectively assessed using photographs collected by researchers (not self-reported intakes by parents) provides important additional insights.

The food classification systems implemented in this study have been used elsewhere, however, there is an element of subjectivity to allocation of foods to non-core and NOVA, which may result in misclassification. We have however used the same NOVA and non-core allocations as previously used in NDNS in an effort to ensure comparability with previous work reporting national intakes^(22, 23)^. Classifications for NOVA were assigned at the food level, not the recipe level, so there may be some misclassification of UPFs. We recommend future studies prospectively collect the processing of food to aid classification.

It was evident from the study that food waste is a key issue for both ECEC provided meals and packed lunches, with approximately 150 kcal extra served than consumed for packed lunches, and 100 kcal extra served than consumed for ECEC provided lunches. The issue of food waste has not been explored in this study, but future analyses to quantify food waste and the resulting cost to both ECEC providers and parents would be beneficial.

The implications of this research are that policy makers at a national level in countries other than Scotland within the UK, and internationally, should explore the opportunities, barriers and cost to reduce packed lunches in ECEC. The lack of differences observed between deprivation status in ECEC provided food suggests further research is warranted in exploring the potential for government funded lunches in reducing health inequalities. The introduction of universal free lunches in ECEC should be urgently considered by policy makers with support from researchers to identify the optimum age or age-range for implementation. Research should also assess the effects on food quantity, quality, waste and cost-effectiveness, as well as the logistics of provision and also the potential for wider benefits which have been found with universal free meals in schools^(48)^. Additionally, studies should explore the context of ECEC provided food consumption by food quality and portion size within the context of the whole day.

Given the similar quality observed between ECEC provided lunches in England and Scotland, and the higher quality of ECEC provided lunches in Scotland compared to packed lunches, it has been demonstrated that it is possible to provide country-wide government funded lunches within ECEC settings that are higher quality than packed lunches. Further investigation is needed into the quality of food provision in Scotland, particularly in relation to compliance with national food standards. In England, new Government early years food guidance for ECEC settings has been published^(33)^ for use from September 2025. Adherence to this guidance should be monitored and its influence on food provision and children’s consumption patterns should be evaluated.

## Conclusion

Packed lunches provided from home in ECEC settings were found to have larger serving sizes and poorer quality compared to ECEC provided meals. Children consuming packed lunches derived a greater proportion of their caloric intake from HFSS foods, as well as from other non-core food items. Furthermore, there was a greater discrepancy between the energy served and the energy consumed among those eating packed lunches relative to children receiving meals provided by ECEC settings suggesting differences in food waste. We also found that food quality in ECEC provided meals did not differ by deprivation, suggesting a potential for reducing health inequalities. Policy makers should consider the potential benefits, challenges and associated costs of increasing the uptake of ECEC provided meals, as well as the introduction of universal free lunches in ECECs. Such policy development would benefit from further research on the optimum age or age-range for universal free lunches and to evaluate the impact on food quantity, quality, waste and cost-effectiveness.

## Supplementary Material

Supplementary Files

This is a list of supplementary files associated with this preprint. Click to download.

• MaishmanetalSUPPLEMENTARYMATERIALSLunchesinUKearlyyearsECECv1.docx

## Figures and Tables

**Table 1:** England and Scotland ECECs: kcal served and consumed, and proportion of kcal served from HFSS, UPF and non-core foods summarised across different child characteristics; estimates from univariable models not provided, but

	Proportion of total kcal served from:										
		Kcal served		Kcal consumed		HFSS foods		UPFs		Non-core foods	
	n/N (%)	Median	IQR	Median	IQR	Median	IQR	Median	IQR	Median	IQR
Overall	532	433.9	(339.9, 564.1)	319.5	(214.4, 439.4)	48.3	(28.2, 66.2)	57.9	(33.7, 79.2)	37.9	(13.2, 57.7)
Age											
24–35 months	100/529 (18.9%)	444.6	(360.6, 540.2)	300.5	(208.1, 419.6)	40.4*	(13.4, 53.0)	47.6	(22.9, 75.4)	30.2	(6.4, 50.9)
36–47 months	264/529 (49.9%)	427.8	(339.5, 585.0)	314.0	(212.8, 442.3)	50.2*	(29.5, 67.6)	60.3	(34.2, 79.7)	40.2	(17.4, 59.7)
48 + months	165/529 (31.2%)	430.1	(331.8, 536.2)	335.1	(234.2, 434.5)	51.6*	(34.3, 68.6)	61.1	(39.7, 80.6)	37.8	(9.4, 57.5)
Gender											
Male	264/529 (49.9%)	429.4	(327.2, 563.5)	300.9	(216.4, 442.9)	50.4	(23.7, 67.8)	55.8	(27.8, 78.8)	35.5	(9.4, 57.9)
Female	265/529 (50.1%)	435.3	(353.8, 565.5)	335.5	(212.1, 431.7)	46.7	(30.5, 65.6)	59.1	(39.1, 79.7)	40.0	(16.8, 57.6)
zBMI											
Healthy weight	370/497 (74.4%)	426.1	(332.8, 566.7)	318.8	(215.5, 428.5)	48.6	(30.5, 66.9)	59.5	(34.6, 79.7)	39.0	(14.4, 57.5)
Unhealthy weight	127/497 (25.6%)	454.3	(350.6, 564.1)	321.2	(196.1, 457.3)	49.0	(26.9, 64.4)	58.0	(35.2, 79.8)	39.5	(16.0, 57.8)
Child deprivation											
High deprivation	190/532 (35.7%)	421.9	(333.0, 530.8)	306.5	(195.7, 409.5)	48.9*	(32.1, 66.6)	57.4	(33.6, 80.3)	38.9	(16.8, 60.2)
Moderate deprivation	233/532 (43.8%)	442.5	(342.1, 572.3)	323.3	(213.8, 445.0)	50.1*	(31.9, 67.7)	60.9	(36.3, 80.3)	38.9	(18.8, 57.2)
Low deprivation	109/532 (20.5%)	432.5	(345.0, 589.7)	351.1	(237.3, 468.5)	42.1*	(15.9, 62.1)	52.8	(22.8, 72.0)	32.5	(4.1, 53.2)
Provider type											
Not attached to school	355/532 (66.7%)	435.2	(340.1, 564.1)	317.3	(207.2, 452.1)	48.0	(18.5, 66.2)	58.7	(28.9, 81.8)	36.7	(8.8, 56.9)
Attached to school	177/532 (33.3%)	432.0	(339.3, 566.0)	320.2	(229.4, 420.5)	49.7	(35.1, 65.6)	57.2	(37.0, 73.4)	39.7	(20.7, 59.9)
Local government area											
Somerset	217/532 (40.8%)	467.2*	(366.8, 602.8)	359.6*	(229.2, 466.6)	47.3	(28.8, 64.4)	61.8	(33.9, 79.9)	42.5	(18.9, 57.5)
Swindon	63/532 (11.8%)	497.7*	(404.0, 588.1)	385.9*	(257.0, 470.3)	52.4	(29.5, 68.2)	58.0	(26.9, 82.9)	41.7	(22.2, 60.2)
Sandwell	79/532 (14.8%)	433.7*	(307.7, 528.9)	231.1*	(170.9, 406.2)	57.2	(34.2, 69.8)	53.1	(29.4, 86.4)	34.2	(16.1, 59.3)
Arran and Ayrshire	173/532 (32.5%)	382.9*	(304.0, 488.0)	287.7*	(201.8, 370.9)	47.2	(21.0, 64.3)	53.1	(36.4, 74.1)	33.6	(0.0, 55.6)
Country											
England	359/532 (67.5%)	466.1*	(362.9, 584.3)	336.6*	(219.7, 454.9)	49.4	(29.5, 66.9)	60.0	(31.3, 80.3)	40.0	(16.8, 58.6)
Scotland	173/532 (32.5%)	382.9*	304.0, 488.0)	287.7*	(201.9, 370.9)	47.2	(21.0, 64.3)	53.1	(36.4, 74.1)	33.6	(0.0, 55.6)
Season of data collection											
Winter/Spring	174/532 (32.7%)	433.1	(355.0, 579.0)	343.2*	(227.3, 452.1)	47.5	(18.4, 66.9)	59.5	(35.5, 80.3)	36.2	(5.8, 58.6)
Summer	174/532 (32.7%)	460.0	(332.8, 556.5)	319.0*	(220.8, 446.7)	51.5	(35.7, 69.5)	61.8	(35.4, 79.8)	38.8	(17.7, 57.5)
Autumn	184/532 (34.6%)	425.5	(329.0, 550.3)	301.8*	(193.6, 410.6)	44.5	(23.5, 63.2)	50.5	(31.5, 76.4)	38.9	(13.1, 55.5)
Lunch provision											
ECEC provided	352/529 (66.5%)	403.3*	(304.7, 523.9)	306.1	(199.4, 413.7)	39.9*	(12.2, 57.5)	46.0*	(23.1, 71.7)	30.1*	(0.0, 52.9)
Packed lunch	177/529 (33.5%)	516.2*	(406.9, 670.8)	359.6	(243.4, 462.8)	61.9*	(46.5, 74.1)	73.1*	(60.0, 86.6)	50.3*	(32.5, 60.8)

Abbreviations: zBMI -standardised body mass index; ECEC - early childhood education and care; HFSS - high fat, salt or sugar; UPF - ultra-processed food; IQR - interquartile range.

* indicate characteristics that showed evidence of association (p < 0.05) with the outcome in univariable analyses.

**Table 2:** Multivariable analyses effect estimates

Model number	Outcome	Main exposure	England and Scotland MD (95% CI), p- value	England only MD (95% CI), p- value	ECEC provided meals only MD (95% CI), p- value
1.1	Total kcal served	Packed lunch*	120.0 (76.6, 163.3), p<0.001	111.2 (60.7, 161.8), p<0.001	-
1.2	Total kcal consumed	Packed lunch*	16.0 (−28.0, 60.0), p = 0.476	9.7 (−38.0, 57.4), p = 0.691	-
1.3a	Proportion of total kcal served from HFSS foods	Packed lunch*	23.9 (17.6, 30.1), p<0.001	26.5 (20.3, 32.8), p<0.001	-
1.3b	Proportion of total kcal consumed from HFSS foods	Packed lunch*	18.2 (11.1,25.3), p<0.001	20.5 (13.3, 27.7), p<0.001	-
1.4a	Proportion of total kcal served from UPFs	Packed lunch*	23.1 (16.1,30.2), p<0.001	28.4 (21.1, 35.8), p<0.001	-
1.4b	Proportion of total kcal consumed from UPFs	Packed lunch*	20.6 (13.2, 27.9), p<0.001	26.2 (18.5, 33.9), p<0.001	-
1.5a	Proportion of total kcal served from non-core foods	Packed lunch*	15.1 (8.8, 21.5), p<0.001	17.9 (11.1, 24.7), p<0.001	-
1.5b	Proportion of total kcal consumed from non-core foods	Packed lunch*	10.5 (3.0, 18.0), p = 0.006	12.2 (4.0, 20.3), p = 0.003	-
2.1	Total kcal served	Scotland**	-		-41.9 (−106.0, 22.1), p = 0.199
2.2	Total kcal consumed	Scotland**	-		-43.6 (−182.2, 95.0), p = 0.538
2.3a	Proportion of total kcal served from HFSS foods	Scotland**	-		5.2 (−5.0, 15.4), p = 0.318
2.3b	Proportion of total kcal consumed from HFSS foods	Scotland**	-		6.2 (−5.5, 17.9), p = 0.298
2.4a	Proportion of total kcal served from UPFs	Scotland**	-		9.7 (−2.2, 21.7), p = 0.110
2.4b	Proportion of total kcal consumed from UPFs	Scotland**	-		10.0 (−1.6, 21.6), p = 0.091
2.5a	Proportion of total kcal served from non-core foods	Scotland**	-		2.6 (−8.2, 13.5), p = 0.636
2.5b	Proportion of total kcal consumed from non-core foods	Scotland**	-		1.2 (−11.8, 14.1), p = 0.859
3.1	Total kcal consumed	Kcal served	0.6 (0.56, 0.69), p<0.001	0.6 (0.5, 0.7), p<0.001	0.8 (0.7, 0.9), p< 0.001
3.2	Total kcal consumed	Proportion of kcal served from HFSS foods	1.4 (0.9, 2.0), p< 0.001	1.3 (0.6, 2.1), p = 0.001	1.8 (1.1,2.5), p< 0.001
3.3	Total kcal consumed	Proportion of kcal served from UPFs	−0.0 (−0.5, 0.5), p = 0.993	−0.1 (−0.8, 0.5), p = 0.726	−0.1 (−0.7, 0.6), p = 0.791
3.4	Total kcal consumed	Proportion of kcal served from non-core foods	0.8 (0.3, 1.4), p = 0.005	0.6 (−0.2, 1.3), p = 0.122	0.8 (0.1, 1.5), p = 0.024

Abbreviations: ECEC - early childhood education and care; HFSS - high fat, salt or sugar; UPF – ultra-processed food; MD – meandifference; CI – confi dence interval.

All models adjusted for Age, Gender, standardised body mass index (zBMI), child deprivation status, provider type and season; area and nursery are fitted as random effects.

* Reference category: ECEC provided lunches

** Reference category: ECEC providers in England

**Table 3:** Mediation analyses for the outcome kcal served

	Model	Outcome	Main exposure	Additional covariates	MD (95% CI), p-value	Mediation effect
Adjusted model	c	Total kcal served	Packed lunch*	-	120.0 (76.6, 163.3), p< 0.001	
HFSS mediation model	a	Proportion of total kcal served from HFSS	Packed lunch	-	23.9 (17.6, 30.1), p< 0.001	
	b	Total kcal served	Proportion of total kcal served from HFSS	-	2.2 (1.6, 2.7), P< 0.001	
	c’	Total kcal served	Packed lunch*	Proportion of total kcal served from HFSS	67.5 (21.5, 113.6), p = 0.004	43.8%
UPF mediation model	a	Proportion of total kcal served from UPFs	Packed lunch*	-	23.1 (16.1, 30.2), p< 0.001	
	b	Total kcal served	Proportion of total kcal served from UPFs	-	0.4 (−0.2, 0.9), p = 0.183	
	c’	Total kcal served	Packed lunch*	Proportion of total kcal served from UPFs	120.2 (74.8, 165.5), p< 0.001	0.0%
Non-core mediation model	a	Proportion of total kcal served from noncore foods	Packed lunch*	-	15.1 (8.8, 21.5), p< 0.001	
	b	Total kcal served	Proportion of total kcal served from noncore foods	-	1.0 (0.4, 1.6), p = 0.001	
	c’	Total kcal served	Packed lunch*	Proportion of total kcal served from noncore foods	108.6 (64.7, 152.4), p < 0.001	9.5%

Abbreviations: ECEC - early childhood education and care; HFSS - high fat, salt or sugar; UPF - ultra-processed food; MD – mean difference; CI confidence interval.

**Table 4:** NDNS main food groups of foods within each combination of food classifications; the five (or more if main food groups had equal prevalence) have been given for each combination.

Food classification	NDNS Main Food Group (all combined)
HFSS, UPF and non-core (n = 169)	Biscuits (24%) Buns, cakes, pastries, & fruit pies (21%) Crisps, and savoury snacks (14%) Other meat, and meat products (4%) Sugar confectionery (4%)
HFSS and UPF, not non-core (n = 45)	Cheese(20%) Coated chicken (11%) Pasta, rice, and other cereals (11%) Yogurt, fromage frais and dairy desserts (9%) Miscellaneous (7%) White bread (7%)
HFSS and non-core, not UPF (n = 36)	Buns, cakes, pastries, & fruit pies (47%) Sugars preserves, and sweet spreads (11%) Biscuits (8%) Meat pies, and pastries (8%) Butter (6%) Pasta, rice, and other cereals (6%) Puddings (6%)
HFSS, not UPF or non-core (n = 26)	Cheese (35%) Fruit (11.5%) Beef, veal, and dishes (8%) Commercial toddlers foods and drinks (8%) Eggs, and egg dishes (8%)
UPF and non-core, not HFSS (n = 23)	Yogurt, fromage frais, and dairy desserts (35%) Soft drinks low calorie (17%) Biscuits (9%) Chips, fried & roast potatoes, and potato products (9%) Miscellaneous (9%)
UPF, not HFSS or non-core (n = 87)	Yogurt, fromage frais, and dairy desserts (15%) Miscellaneous (14%) Puddings (11%) Cooked vegetables (11%) White bread (10%)
Non-core, not HFSS or UPF (3)	Chips, fried & roast potatoes and potato products (33%) Other meat and meat products (33%)
Not HFSS, UPF or non-core (n = 162)	Cooked vegetables (22%) Fruit (19%) Salad and other raw vegetables (10%) Pasta rice and other cereals (9%) Chicken and turkey dishes (6%) Miscellaneous (6%) Other potatoes potato salads & dishes (6%)

Abbreviations: NDNS - National Diet and Nutrition Survey; HFSS - high fat, salt or sugar; UPF – ultra-processed food.

## Abbreviations

ECEC: early childhood education and care
HFSS: high fat, salt or sugar
UPF: ultra-processed food
